# A Transcontinental Pilot Study for Acupuncture Lifting-Thrusting and Twisting-Rotating Manipulations

**DOI:** 10.1155/2012/157989

**Published:** 2012-12-01

**Authors:** Tao Huang, Weibo Zhang, Shuyong Jia, Yuying Tian, Guangjun Wang, Lijian Yang, Ingrid Gaischek, Lu Wang, Gerhard Litscher

**Affiliations:** ^1^Institute of Acupuncture and Moxibustion, China Academy of Chinese Medical Science, No. 16 Nanxiaojie of Dongzhimen, Beijing 100700, China; ^2^Beijing Key Laboratory of Traditional Chinese Veterinary Medicine, Beijing University of Agriculture, Beijing 102206, China; ^3^Stronach Research Unit for Complementary and Integrative Laser Medicine, Research Unit of Biomedical Engineering in Anesthesia and Intensive Care Medicine, TCM Research Center Graz, Medical University of Graz, Auenbruggerplatz 29, 8036 Graz, Austria

## Abstract

The goal of this study was to observe possible changes of the skin microvascular perfusion on the acupoints and related areas and to quantify influences of acupuncture stimulation on the volunteers' blood pressure, heart rate, and heart rate variability (HRV). During the measurement, the needling sensations of volunteers were enquired and recorded. Ten healthy volunteers with a mean age ± SD of 25.4 ± 2.6 years were enrolled, and acupuncture stimulation was performed on ST36 (Zusanli, right side), in pure lifting-thrusting or twisting-rotating manipulation. During needling, we observed the changing of microvascular perfusion on ST36, 37, 38, and a control point using MOOR speckle laser blood flow scanning. Electrocardiogram and blood pressure were registered before, during, and after needling. Both lifting-thrusting and twisting-rotating needle manipulations could decrease blood pressure and heart rate while improving HRV significantly. There were significant differences in microvascular perfusion on acupoints ST36, 37, 38, and the control point following these two kinds of needle manipulation. The needling sensation caused by lifting-thrusting is stronger than that of twisting-rotating manipulation. Significant differences between lifting-thrusting and twisting-rotating acupuncture stimulation methods show that the mechanisms may be different and need to be researched thoroughly in the future.

## 1. Introduction and Background

In clinical practice, the medical acupuncturist always stimulates the patients using compound manipulations including lifting, thrusting, twisting, and rotating. Lifting-thrusting (*提插*) and twisting-rotating (*捻转*) are the basic hand needling operations. The former means that the acupuncturist inserts the needle into the skin to a certain depth to obtain the qi arrival and then moves the needle up and down perpendicularly to stimulate the tissues including skin, fascia, fat, and muscles [1, 2]. The latter means that the acupuncturist inserts the needle into the skin to a certain depth to obtain the qi arrival then moves the needle horizontally rotating. In general, the stimulus intensity caused by the rotary motion with an angle less than 360 degrees is smaller than that of lifting-thrusting. If the rotary angle exceeds 360 degrees, the moving needle will twine the muscle fiber and cause a stronger stimulation, even an uncomfortable feeling for the patients [2, 3]. This phenomenon must be avoided in clinical practice.

There are not many studies of pure lifting-thrusting and twisting-rotating in the scientific literature [4]. Wang [5, 6] studied the acupuncture process from the angle of energy; he found that the lifting-thrusting method yields a comparatively larger energy input than the twisting-rotating method; however, the difference was not statistically significant. The speed of manipulation seems to have more influence on energy transmission along the channels [5].

Our previous studies show that acupuncture stimulation on certain acupoints can change the blood flow of neighboring points/areas of the same channel significantly. If stimulation is performed in different ways, the recorded changes in biosignals are also different [7]. Therefore, we designed and carried out this pilot experiment for studying skin microvascular perfusion on an acupoint, its neighboring area, and nonacupoints.

### 1.1. Research Objective

We observed the changes in skin microvascular perfusion on acupoints and the corresponding areas caused by pure lifting-thrusting and twisting-rotating stimulations, as well as volunteers' blood pressure, heart rate (HR), and heart rate variability (HRV) so as to investigate the relationship among acupoints, non-acupoints, and the local points on the same meridian. During the measurement, the needling sensations were inquired and recorded. Analysis of HR and HRV in this partially single-blind transcontinental study was performed in Europe.

## 2. Methods

### 2.1. Healthy Volunteers

Ten healthy volunteers with a mean age ± SD of 25.4 ± 2.6 years (8 female, 2 male), all from Beijing University of Traditional Chinese Medicine and Graduate School of China Academy of Chinese Medical Sciences, were enrolled in this study. The volunteers had no medical history whatsoever during the last year. All of them had had experience of acupuncture before, so they could accurately describe the needling sensation.

### 2.2. Acupuncture Operations

Using single-use sterile needles (Huacheng Brand, Suzhou, China), 0.30 (diameter) × 40 (length) mm, we punctured Zusanli (ST36, right side), which is located in the lower limb, one finger's breadth from the anterior crest of the tibia, 3 cun below the knee joint [8]. All acupuncture operations were performed by a specialized acupuncturist in China.

#### 2.2.1. Lifting-Thrusting

**Figure 1:**
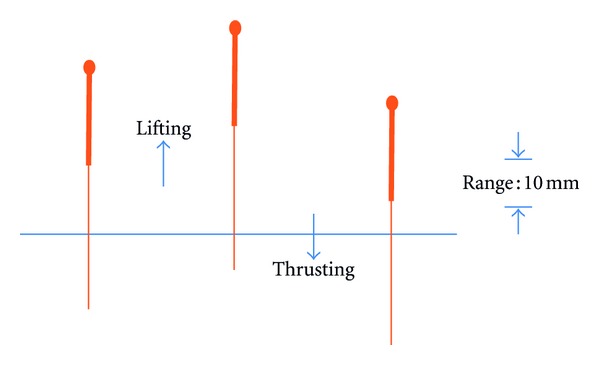
Lifting-thrusting needle manipulation.

The needle was inserted to a certain depth till the acupuncturist felt qi arriving, asking the volunteer whether she/he felt any needling sensations at the same time. After 5 minutes scanning, the lifting-thrusting manipulation was done for 20 seconds, with a range of 10 mm, frequency 1–1.5 Hz (see Figure 1).

#### 2.2.2. Twisting-Rotating

**Figure 2:**
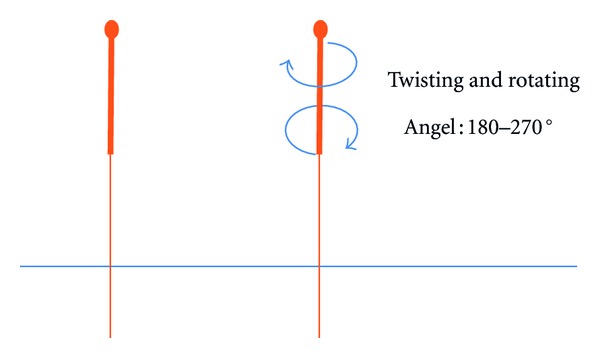
Twisting-rotating needle manipulation.

The needle was inserted to a certain depth till the acupuncturist felt qi arriving, asking the volunteer whether she/he felt any needling sensations at the same time. After 5 minutes scanning, the twisting-rotating manipulation was done for 20 seconds, with an angle of 180–270°, frequency 2–2.5 Hz (see Figure 2).

### 2.3. Instruments and Measurement Methods

**Figure 3:**
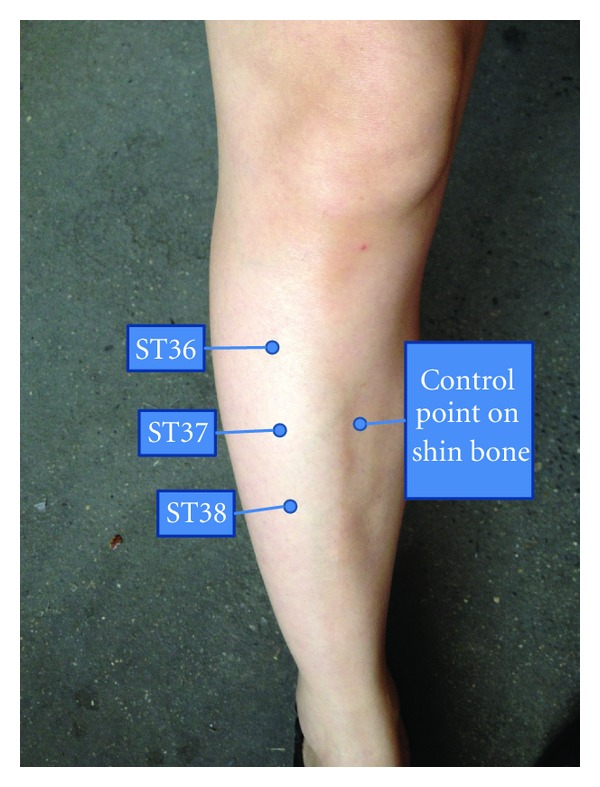
Point diagram.

For visualization of microvascular changes a multipoint synchronization scanner Moor FLPI (Moor Instruments Ltd., Millwey, UK) was used. The scanning distance was about 11 cm. The acupoints ST36, 37, and 38 (see Figure 3) were marked, as well as the control point (at the same level with ST37, on the tibia crest; touching the bone could help to affirm the point).

**Figure 4:**
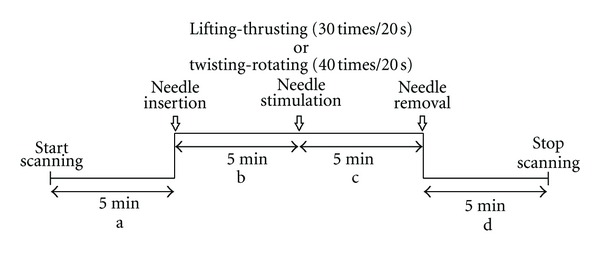
Scanning program diagram.

The temperature of the laboratory was kept constant at 26°C, and the volunteers were asked to come into the room 5 minutes ahead of schedule to adapt to the room temperature. During the scanning procedure (see Figure 4), the volunteers also underwent electrocardiographic (HR, HRV) and blood pressure monitoring. An HRV recorder MedilogAR12 (Huntleigh Healthcare, Cardiff, UK; partially developed in Austria and provided by the Medical University of Graz) and a hematomanometer OMRON HEM-7112 (OMRON Corp., Tokyo, Japan) were used. In addition, the volunteers were asked to assess their needling sensations by visual analogue scale (VAS) after needling before manipulating, after manipulating the needle, and after removing the needle. “0” means “no sensation at all” and “10” means “the needling sensation is too strong to bear.” 

### 2.4. Statistical Analysis

The alterations of the skin microvascular perfusion were transferred into measurement data by the MoorVer2.0 software (Moor Instruments Ltd., Millwey, UK). All data including blood pressure, HR, and needling sensation (VAS) were analyzed using Friedman repeated measures ANOVA on ranks. In addition, Tukey test as post hoc analysis and the *t*-test, with *P* < 0.05 defined as the level of significance, were used. The HRV data were analyzed at the laboratory in Austria, and the analyzed results were sent back to Beijing. The researchers in Austria did not know the type of needle manipulation.

## 3. Results

### 3.1. Influence of BP and HR Caused by Different Needling Manipulations

The results show that systolic blood pressure (SBP), diastolic blood pressure (DBP), and HR decreased significantly after lifting-thrusting manipulation, but only SBP and HR decreased significantly after twisting-rotating manipulation while DBP increased (see Table 1).

### 3.2. Influence of Different Needling Manipulations on Skin Microvascular Perfusion on the Acupoint and Its Corresponding Area

Among the 4 observed points, skin blood perfusion of the acupoint ST36 increased significantly after acupuncture needle insertion before lifting-thrusting needle manipulation, after manipulation and after removing the needle; the skin blood perfusion of the control point on the tibia bone decreased insignificantly. Skin blood perfusion of ST37 and ST38 changed inconspicuously (Table 2).

During the twisting-rotating needling manipulation, the skin blood perfusion of ST36 increased and reached its maximum, then decreased significantly step by step. Point ST37 showed similar changes. At ST38 and the control point, the skin blood perfusion decreased. But only the increase at ST37 after removing the needle and the decrease at ST38 after twisting-rotating manipulation was statistically significant (Table 3).

### 3.3. Influence of Different Needling Manipulations on HRV

**Figure 5:**
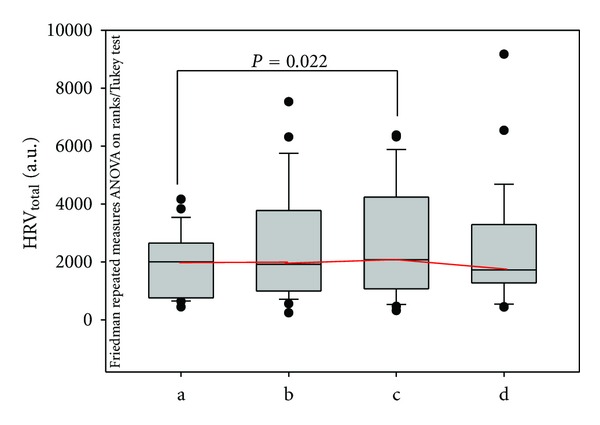
Box plot presentation of total heart rate variability. The line in the box indicates the median, the ends of the boxes define the 25th and 75th percentile, the error bars represent the 10th and 90th percentile, respectively, and the points represent the outliers.

As mentioned in Section 2, HRV was also analyzed.  Figure 5 shows the results from the analysis of total HRV. There was a significant increase in HRV at the end of the stimulation procedure (phase c). A separate analysis of the different stimulation methods did not show any significant alterations in the ten volunteers.

### 3.4. Needling Sensation Caused by Different Needling Manipulations

**Figure 6:**
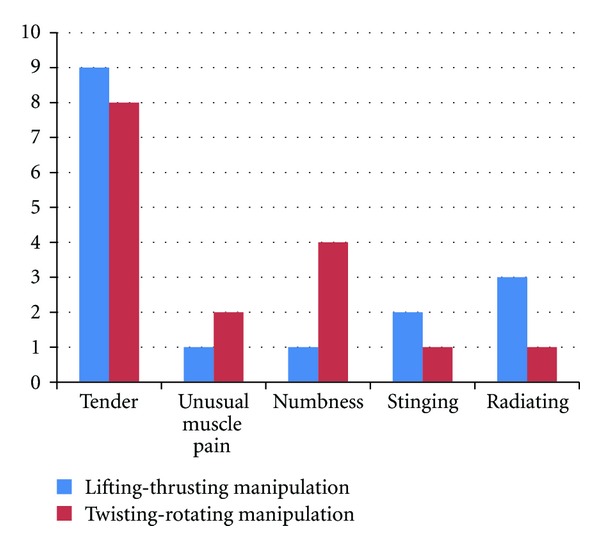
The different needling sensations of the 10 healthy volunteers caused by the two kinds of needle manipulation.

**Figure 7:**
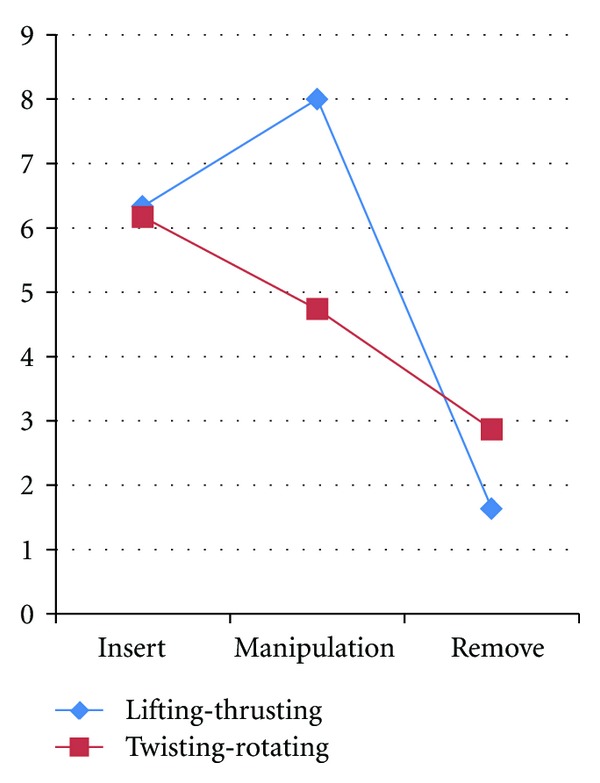
VAS-score of the needling manipulations.

According to the questionnaires about the needling sensation, we found that the main feelings after inserting or removing the needle were tenderness and numbness (Figure 6), and there was no difference in intensity. After manipulation, the needling sensation caused by lifting-thrusting was significantly stronger than that of twisting-rotating (Figure 7).

## 4. Discussion

Quantifying acupuncture needle manipulation using modern techniques and measurement methods has been performed only by few research groups [9]. There are, for example, several investigations concerning force parameters which can be quantified in different settings. Relationships between displacements and rotation frequencies, as well as between displacement and force amplitudes showed considerable variability across individual acupuncturists and subjects [9–14].

Needle manipulations and arrival of qi (needling sensation) are among the most important aspects in acupuncture. Needling manipulation, also known as needling transmission, refers to various manipulations of acupuncture to induce needling sensation after the needle is inserted. The arrival of qi, also known as needling sensation, refers to induction of channel qi after the needle is inserted. During this needling sensation, the patient feels numbness, soreness, or heaviness around the acupuncture point. Sometimes it is connected with coldness, warmness, itching, pain, electric shock feeling, ant crawling, feeling, and so on [8]. The fundamental manipulation techniques can be divided into two main types: lifting and thrusting, and twirling or rotating. These two techniques usually may be used either alone or in combination, according to the patient's clinical condition [8]. To the best of our knowledge, there are no investigations concerning changes of microcirculation and other cardiovascular and neurovegetative parameters during different kinds of acupuncture needle manipulations. In this first transcontinental pilot study, we found significant changes between the two main manipulation methods, even though the group of healthy volunteers investigated was very small. Our investigations clearly showed that there is no change of microcirculatory parameters at a control point, however, there were significant changes at acupoints located on the same meridian as the stimulated one (ST36). In addition, it could be shown that blood pressure and heart rate changed manipulation-related changes.

## 5. Conclusion

Both lifting-thrusting and twisting-rotating needling manipulations could effectively decrease BP and HR and improve HRV. However, the results were not completely uniform; they showed that the mechanisms are different and worth being studied deeply. In order to further elucidate these mechanisms, the results should also be compared with those of other modes of stimulation at ST 36, such as electroacupuncture, laser acupuncture, and moxibustion in future studies.

In this experiment, the manipulation of twisting-rotating completely copied the clinical operation, the angle of rotation being smaller than 360°. Therefore, the needling sensation caused by lifting-thrusting manipulation was stronger than that of twisting-rotating.

The control point showed a continuous decrease in skin blood perfusion, while the acupoints ST36, 37, and 38 all showed significant increases. This may indicate that there could be a specificity of channels and acupoints.

## Figures and Tables

**Table 1:** The influence of lifting-thrusting or twisting-rotating manipulation on BP and HR.

	Lifting-thrusting		Twisting-rotating	
	Before acupuncture	After acupuncture	Before acupuncture	After acupuncture
SBP (mmHg)	117.87 ± 8.37	106.67 ± 7.75**	112.67 ± 10.26	106.53 ± 9.52**
DBP (mmHg)	69.33 ± 8.93	65.87 ± 8.61*	64.8 ± 8.16	65.4 ± 8.55
HR (1/min)	73.9 ± 9.9	68.9 ± 6.0**	78.9 ± 13.3	74.3 ± 10.3**

*P < 0.01; **P < 0.001.

**Table 2:** The changes in skin microvascular perfusion (in arbitrary units) at all investigated points during lifting-thrusting needling manipulation.

	Before acupuncture	Before lifting-thrusting manipulation	After lifting-thrusting manipulation	After removing the needle
ST36	44.71 ± 7.14	72.96 ± 5.16**	81.99 ± 0.50*	82.23 ± 9.41**
ST37	51.79 ± 24.33	52.76 ± 38.61	50.58 ± 29.63	48.82 ± 27.01
ST38	48.57 ± 18.81	48.04 ± 25.03	46.4 ± 26.52*	45.86 ± 28.64
Control point	56.65 ± 16.33	52.87 ± 21.28	51.13 ± 15.84	50.27 ± 14.57

*P < 0.01; **P < 0.001.

**Table 3:** The changes of skin microvascular perfusion (in arbitrary units) at all investigated points during twisting-rotating needling manipulation.

	Before acupuncture	Before twisting-rotating manipulation	After twisting-rotating manipulation	After removing the needle
ST36	37.63 ± 33.64	57.54 ± 56.50**	52.87± 25.68**	50.08 ± 48.86*
ST37	47.84 ± 14.12	50.32 ± 16.38	48.69 ± 16.21	45.79 ± 14.47**
ST38	45.5 ± 13.50	44.7 ± 14.53	43.42 ± 13.25*	42.44 ± 13.36*
Control point	49.19 ± 17.42	47.97 ± 17.27	47.51 ± 16.79	45.59 ± 14.78

*P < 0.01; **P < 0.001.
